# Indirect Comparison of Neoadjuvant Treatment Strategies for Muscle‐Invasive Bladder Cancer: ddMVAC and Perioperative Durvalumab‐Gemcitabine‐Cisplatin Versus Gemcitabine‐Cisplatin: A Systematic Review and Network Meta‐Analysis of Randomised Controlled Trials

**DOI:** 10.1002/ijc.70525

**Published:** 2026-04-28

**Authors:** Maurin Helen Mangold, Gloria Baumann, Victoria Luise Simone Wieland, Nicolas Carl, Luisa Vivienne Renner, Alexander Studier‐Fischer, Hanna Menold, Stefanie Zschäbitz, Frederik Wessels, Niklas Westhoff, Maurice Stephan Michel, Karl‐Friedrich Kowalewski, Caelán Max Haney‐Aubert

**Affiliations:** ^1^ Department of Urology and Urosurgery, University Medical Center Mannheim Medical Faculty Mannheim at Heidelberg University Mannheim Germany; ^2^ Division of Intelligent Systems and Robotics in Urology (ISRU) German Cancer Research Center (DKFZ) Heidelberg Heidelberg Germany; ^3^ DKFZ Hector Cancer Institute at the University Medical Center Mannheim Mannheim Germany; ^4^ Department of Medical Oncology, National Centre for Tumour Diseases (NCT) University Hospital Heidelberg Heidelberg Germany

**Keywords:** muscle‐invasive bladder cancer, neoadjuvant chemotherapy, network meta‐analysis

## Abstract

For cisplatin‐eligible patients with muscle‐invasive bladder cancer (MIBC) undergoing radical cystectomy (RC), neoadjuvant cisplatin‐based chemotherapy (NAC) is standard of care. More intensive regimens such as dose‐dense methotrexate, vinblastine, doxorubicin and cisplatin (ddMVAC) and chemoimmunotherapy with durvalumab plus gemcitabine‐cisplatin (D‐GC) have shown superior outcomes. This network meta‐analysis (NMA) compares the efficacy of ddMVAC, D‐GC and GC and explores the efficacy thresholds for emerging therapy options, such as enfortumab vedotin plus pembrolizumab (EV‐P), to surpass current standards. Following PROSPERO registration (CRD420251077606), systematic searches of PubMed, CENTRAL and Web of Science were conducted to March 2025. Randomised controlled trials (RCTs) comparing neoadjuvant regimens in cisplatin‐eligible MIBC were included. A random‐effects NMA was performed. Simulations explored hypothetical hazard ratios (HRs) for EV‐P. Three RCTs were included. ddMVAC (HR 0.75, 95% CI 0.58–0.98; *p* = 0.034) and D‐GC (HR 0.75, 95% CI 0.66–0.85; *p* < 0.001) improved overall survival (OS) versus GC, without differences between ddMVAC and D‐GC (HR 0.99, 95% CI 0.75–1.33; *p* = 0.97). Both regimens improved progression‐free survival. D‐GC achieved higher pathological complete response (pCR) versus GC (OR 1.57, 95% CI 1.21–2.03; *p* < 0.001), whereas ddMVAC did not. No significant difference in pCR was found between ddMVAC and D‐GC. Simulation‐NMA suggested EV‐P would need to achieve HR ≤ 0.45 versus GC to outperform ddMVAC and D‐GC. Limitations include few trials and indirect comparisons. DdMVAC and D‐GC improve survival compared with GC in neoadjuvant MIBC. Alternative therapeutic strategies must demonstrate substantial survival benefits to warrant replacing established neoadjuvant regimens.

## What's New?

Optimizing neoadjuvant therapy for muscle‐invasive bladder cancer patients who are eligible for cisplatin remains difficult, owing particularly to high toxicity and recurrence rates. This meta‐analysis compared dose‐dense methotrexate, vinblastine, doxorubicin, and cisplatin (ddMVAC) against durvalumab plus gemcitabine‐cisplatin (D‐GC) and GC alone, using simulations to estimate efficacy thresholds for emerging therapies. Both ddMVAC and D‐GC improved survival versus GC. D‐GC showed higher complete response. Simulations indicate that enfortumab vedotin plus pembrolizumab, an emerging immunotherapy combination, would require substantial efficacy gains to outperform existing regimens. The findings support ddMVAC and D‐GC as current standards and provide benchmarks for evaluating novel therapies.

## Graphical abstract

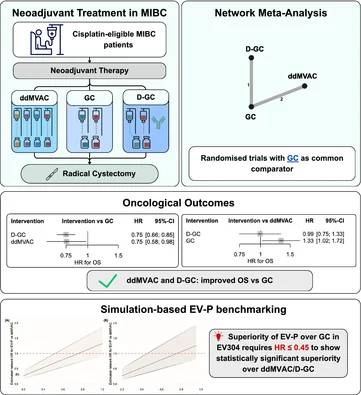

**Abbreviations**
ADC: antibody drug conjugate
CENTRAL: Cochrane Central Register of Controlled Trials
CI: confidence interval
CTCAE: Common Terminology Criteria for Adverse Events
ctDNA: circulating tumour DNA
ddMVAC: dose‐dense methotrexate, vinblastine, doxorubicin and cisplatin
df: degrees of freedom
D‐GC: durvalumab plus gemcitabine‐cisplatin
EV‐P: enfortumab vedotin plus pembrolizumab
GC: gemcitabine‐cisplatin
GRADE: Grading of Recommendations, Assessment, Development and Evaluation
GRADEpro: GRADEpro software for GRADE evidence profiles
HR: hazard ratio
*I* ^2^
: inconsistency (heterogeneity) statistic
MIBC: muscle‐invasive bladder cancer
MVAC: methotrexate, vinblastine, doxorubicin (adriamycin) and cisplatin
NAC: neoadjuvant chemotherapy
NCI: National Cancer Institute
NMA: network meta‐analysis
OR: odds ratio
OS: overall survival
pCR: pathological complete response
PFS: progression‐free survival
PICOS: Population, Intervention, Comparator, Outcomes, Study design
PRISMA: Preferred Reporting Items for Systematic Reviews and Meta‐Analyses
PROSPERO: International Prospective Register of Systematic Reviews
QoL: quality of life
RC: radical cystectomy
RCT: randomised controlled trial
REML: restricted maximum‐likelihood method
RoB: risk of bias
RoB‐2: Cochrane Risk of Bias 2 tool
SD: standard deviation
SOC: standard of care
SoF: Summary of Findings
TRAE: treatment‐related adverse event
utDNA: urinary tumour DNA

## 1 Introduction

Muscle‐invasive bladder cancer (MIBC) remains a significant clinical challenge due to its high recurrence and cancer‐specific mortality rates [1]. For patients who are eligible for cisplatin, the standard of care is neoadjuvant cisplatin‐based chemotherapy (NAC), which has been shown to improve overall survival (OS) in multiple randomised controlled trials (RCTs) [2, 3, 4]. Although current international guidelines strongly recommend NAC for eligible MIBC patients, real‐world data show that only about 30% receive it [5]. Among NAC regimens, gemcitabine‐cisplatin (GC) has become the most widely used due to its manageable safety profile and broad applicability [6, 7]. Recent RCTs (VESPER, SWOG S1314 and NIAGARA) have evaluated the efficacy of alternative regimens and have shaped the ongoing debate on optimising neoadjuvant and perioperative therapy in MIBC [8, 9, 10]. The VESPER trial investigated an intensified chemotherapy regimen, dose‐dense methotrexate, vinblastine, doxorubicin and cisplatin (ddMVAC), comparing it to standard GC. The study included patients in both the neoadjuvant and adjuvant settings, with approximately 90% receiving NAC [9]. SWOG S1314, primarily designed as a biomarker study, also examined ddMVAC versus GC, but in a purely neoadjuvant context with lower cumulative ddMVAC intensity, administering only four cycles versus six in VESPER [10]. The NIAGARA trial expanded the therapeutic paradigm by combining GC with perioperative immunotherapy, introducing durvalumab (D‐GC) before and after surgery, thereby adding an immunomodulatory and perioperative component to the conventional NAC framework. While all three trials provide crucial evidence, the absence of direct comparisons between ddMVAC and chemoimmunotherapy currently leaves clinicians without clear guidance in selecting between these competing strategies.

To inform clinical decision making, we conducted a network meta‐analysis (NMA) to indirectly compare ddMVAC and D‐GC. Additionally, to contextualise our findings within the landscape of emerging immunotherapy combinations in the neoadjuvant and perioperative setting—specifically enfortumab vedotin plus pembrolizumab (EV‐P), which is currently being investigated in the EV‐304 trial—we conducted a simulation‐based NMA.

This NMA provides a comparative framework to support the selection of evidence‐based neoadjuvant treatments for MIBC, bridging the gap between existing trials and anticipating future developments in this evolving field.

## 2 Materials and Methods

This NMA offers a comparative analysis of studies examining neoadjuvant systemic treatment strategies for patients with MIBC. Adjuvant‐only trials were excluded. The review was conducted in accordance with the guidelines of the Preferred Reporting Items for Systematic Reviews and Meta‐Analyses (PRISMA), the Cochrane Handbook for Systematic Reviews of Interventions and the protocols of the Study Centre of the German Society for Surgery [11, 12]. The research plan was formally documented and officially registered with PROSPERO (CRD420251077606) prior to the initiation of the study.

### 2.1 Search Strategy

**FIGURE 1:**
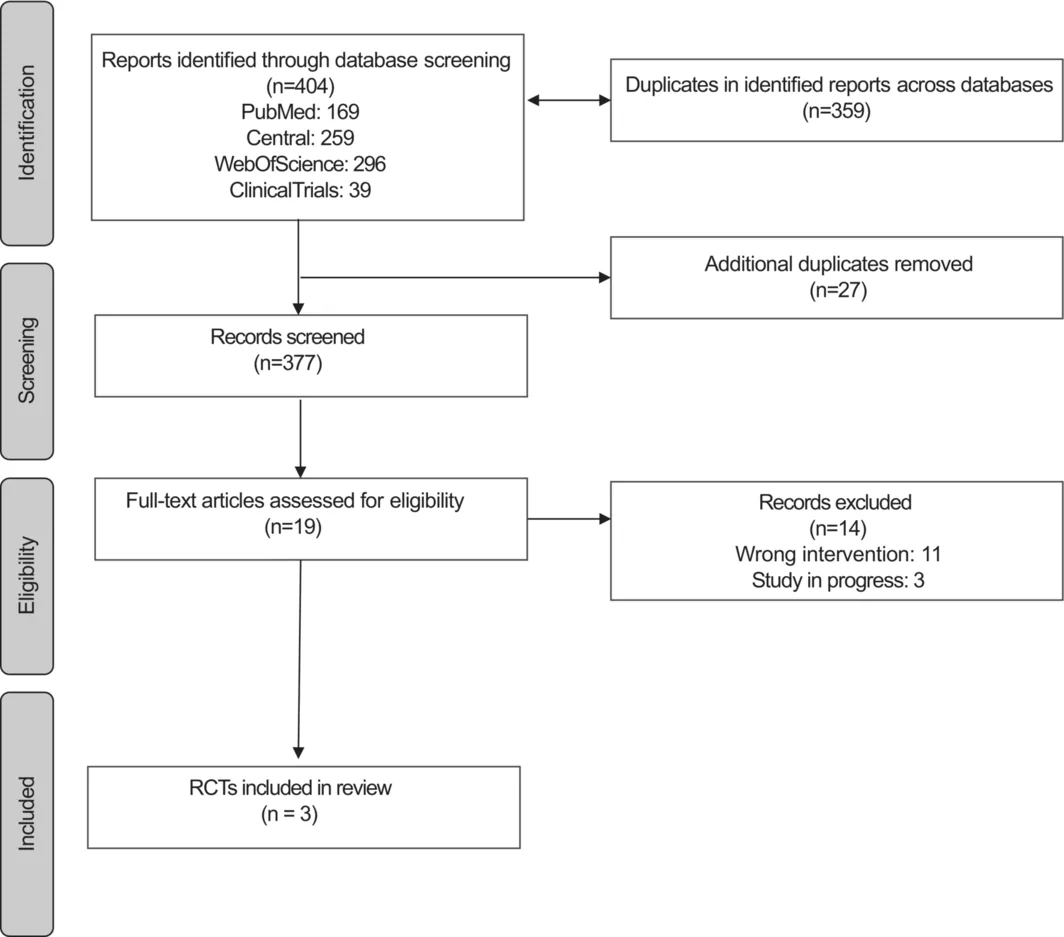
PRISMA flow diagram of the systematic literature search and study selection process.

In accordance with the recommendations set out by Goossen et al. [13], a systematic search was conducted across multiple electronic databases, namely Cochrane CENTRAL, PubMed and Web of Science. The most recent search was updated March 2025. A search for ongoing trials was conducted using ClinicalTrials.gov. The present study exclusively incorporates English‐language articles. The search followed the PICOS framework; the full search strategy is described in detail in Figure 1 [14].

*The following PICOS criteria were used*:

**Table:** 

P (patients)	Patients aged > 18 years with MIBC (staged cT2‐T4a, N0‐N1, M0) who are eligible for cisplatin‐based chemotherapy and scheduled for RC
I (intervention)	1. Neoadjuvant ddMVAC
	2. Neoadjuvant GC + perioperative immune checkpoint inhibitor (e.g., durvalumab, atezolizumab, nivolumab and pembrolizumab)
	3. Perioperative enfortumab vedotin and pembrolizumab (EV‐P)
C (comparator)	1. Neoadjuvant GC
	2. Any direct comparison between interventions (e.g., EV‐P vs. ddMVAC, or ddMVAC vs. GC + immune checkpoint inhibitor)
O (outcomes)	Primary outcome: OS
	Secondary outcomes (if reported): Pathologic complete response (pCR), progression‐free survival (PFS), treatment‐related adverse events (TRAE)
S (study design)	Randomised controlled trials (RCTs). Both completed and ongoing trials are eligible

### 2.2 Data Collection and Analysis

Two reviewers (C.M.H. and M.H.M.) independently assessed the titles, abstracts and full texts of the articles identified in the literature search to determine their eligibility based on inclusion criteria. There was no disagreement in the selection of the identified articles; thus, a third reviewer was not required. References were imported into EndNote 20 (Clarivate Analytics, London, UK) and duplicates were removed. The study characteristics were reviewed and reported for each included study.

### 2.3 Assessment of Risk of Bias and Certainty of Evidence

Risk of bias assessment was independently performed by the two reviewers by applying the RoB‐2 tool for randomised trials [15]. Both reviewers then evaluated the certainty of evidence using GRADEpro Software (McMaster University and Evidence Prime Inc., Ontario, Canada) [16]. Disagreements were resolved through consensus.

### 2.4 Statistical Analysis

Dichotomous outcomes were documented by recording event counts and total number of participants. Means (*M*) and standard deviations (SD) were recorded for continuous outcomes. Missing values (e.g., means and SDs) were imputed using standard methods from the Cochrane Handbook for Systematic Reviews of Interventions [11]. The NMA was conducted using the netmeta package (version 2.8‐0) in R (version 4.1.1). Effect estimates were reported as hazard ratios (HRs) for time‐to‐event (OS, progression‐free survival [PFS]) and as odds ratios (ORs) for binary endpoints (pathological complete response [pCR] and treatment‐related adverse events ≥ grade 3 [TRAE]). Estimates were obtained using random‐effects models with restricted maximum‐likelihood method (REML) for HRs or DerSimonian‐Laird for ORs. Statistical heterogeneity was quantified using *τ* ^2^ and *I* ^2^ statistics. For each endpoint, league tables, forest plots and network diagrams were generated; statistical significance was defined as a *p*‐value ≤ 0.05.

For the hypothetical positioning of EV‐P versus GC a simulation framework was developed to integrate EV‐P into the existing network. Assumed HRs for EV‐P versus GC were varied from 0.20 to 1.00 in increments of 0.05. Event counts for the GC arm were estimated at 0.007 events per patient/month based on the event rates on the included trials for the other comparisons (VESPER, NIAGARA and SWOG 1314). For each scenario, the corresponding standard error was calculated using the inverse‐variance method, and EV‐P was incorporated as an additional treatment node. The NMA was iteratively recalculated at both 2‐ and 5‐year timepoints to estimate the network‐derived HRs for EV‐P versus ddMVAC and D‐GC. Statistical significance was defined both by *p* ≤ 0.05 and by 95% confidence intervals not crossing 1.0.

## 3 Results

### 3.1 Study Characteristics

A total of 404 articles were identified through the systematic search. Following title and abstract screening, 19 full‐text articles were reviewed for eligibility. Ultimately, 3 studies met inclusion criteria and were incorporated into this NMA [8, 10, 17, 18, 19]. A detailed overview of the study selection process is provided in the PRISMA flow diagram (Figure 1). Study characteristics are described in Table 1.

**TABLE 1:** Baseline patient characteristics of the included RCTs for the NMA.

Study	Arm	*n*	Age (median)	ECOG 0 (*n*/%)	ECOG 1 (*n*/%)	≤ T2 (*n*/%)	> T2 (*n*/%)	N0 (*n*/%)	N1 (*n*/%)	Mean follow up (OS; months)
Flaig 2023 S1314	ddMVAC	112	64.8	86 (76.8%)	26 (23.2%)	98 (87.5%)	14 (12.5%)	112 (100.0%)	0 (0.0%)	46.3
	GC	115	64.9	88 (76.5%)	27 (23.5%)	102 (88.7%)	13 (11.3%)	115 (100.0%)	0 (0.0%)	46.3
Pfister 2022 VESPER	ddMVAC	248 (218 NAC)	63	165 (67%)	82 (33%)	197 (90.4%)	21 (9.6%)	218 (100.0%)^a^	0 (0.0%)^a^	63.6
	GC	245 (219 NAC)	63	171 (70%)	72 (29%)	207 (94.5%)	12 (5.5%)	219 (100.0%)^a^	0 (0.0%)^a^	63.6
Powles 2024 NIAGARA	D‐GC	533	65	418 (78.4%)	115 (21.6%)	215 (40.3%)	318 (59.7%)	505 (94.7%)	28 (5.3%)	40
	GC	530	66	415 (78.3%)	115 (21.7%)	213 (40.2%)	317 (59.8%)	500 (94.3%)	30 (5.7%)	40

^a^ Data obtained from the NAC subgroup of the VESPER trial.

### 3.2 Overall Survival

**FIGURE 2:**
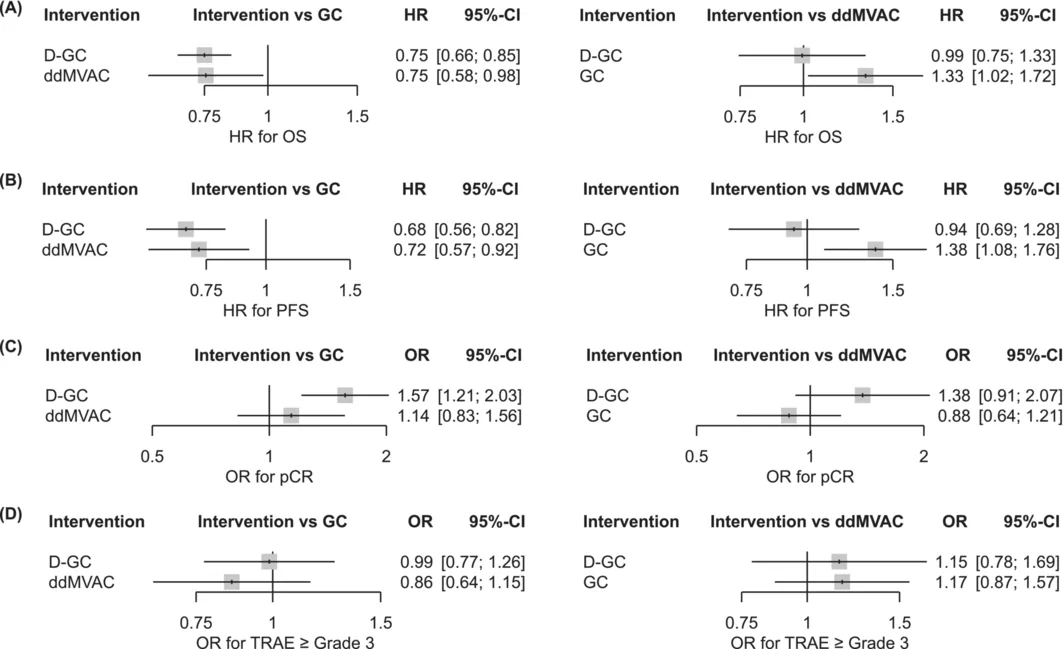
Forest plots of network meta‐analysis results for all four endpoints. Each panel displays two forest plots: Interventions versus GC (left) and versus ddMVAC (right). (A) Overall survival (HR < 1 favours the intervention). (B) Progression‐free survival (HR < 1 favours the intervention). (C) Pathological complete response rate (OR > 1 favours the intervention). (D) Treatment‐related adverse events ≥ grade 3 (OR < 1 favours the intervention). Squares represent point estimates; horizontal lines represent 95% confidence intervals. The vertical reference line indicates no treatment effect (HR or OR = 1.0). CI, confidence interval; ddMVAC, dose‐dense methotrexate, vinblastine, doxorubicin and cisplatin; D‐GC, durvalumab plus gemcitabine‐cisplatin; GC, gemcitabine‐cisplatin; HR, hazard ratio; OR, odds ratio.

The NMA included three neoadjuvant and perioperative strategies, ddMVAC, D‐GC and GC, across two trial designs and three pairwise comparisons, with GC serving as the common comparator (Figure S1). All three RCTs reported OS data, with median follow‐up times ranging from 40 to 64 months. In the random‐effects model, ddMVAC versus GC yielded an OS HR of 0.75 (95% CI 0.58–0.98; *p* = 0.034) and D‐GC versus GC an OS HR of 0.75 (95% CI 0.66–0.85; *p* < 0.001) (Figure 2). The indirect comparison between D‐GC and ddMVAC yielded an HR of 0.99 (95% CI 0.75–1.33; *p* = 0.97), indicating no statistically significant difference between the two interventions. Treatment ranking based on *P*‐scores showed that D‐GC ranked highest for OS (*P*‐score = 0.758), followed closely by ddMVAC (*P*‐score = 0.733), while GC ranked lowest (*P*‐score = 0.009). Heterogeneity was low (*I* ^2^ = 0%, *τ* ^2^ < 0.0001) and there was no evidence of inconsistency (*Q* = 0.49, df = 1, *p* = 0.48). Between‐design inconsistency could not be assessed due to network structure.

### 3.3 Progression Free Survival and Pathological Complete Response

In the random‐effects NMA, both ddMVAC (HR 0.72, 95% CI 0.57–0.92; *p* = 0.009) and D‐GC (HR 0.68, 95% CI 0.56–0.82; *p* < 0.001) significantly improved PFS compared to GC (Figure 2). Indirect comparison between D‐GC and ddMVAC yielded no statistically significant difference in PFS (HR 0.94, 95% CI 0.69–1.28; *p* = 0.69). Treatment ranking based on *P*‐scores for PFS resulted in the following: D‐GC (0.827), ddMVAC (0.670) and GC (0.002). Between‐design inconsistency and within‐design heterogeneity were negligible (*τ* ^2^ < 0.0001, *I* ^2^ = 0%, *Q* = 0.11, *p* = 0.74). In the NMA examining pCR, D‐GC was associated with significantly higher odds of response compared to GC (OR 1.57, 95% CI 1.21–2.03; *p* < 0.001), whereas ddMVAC showed no statistically significant difference compared to GC (OR 1.14, 95% CI 0.83–1.56; *p* = 0.42) (Figure 2). The indirect comparison between ddMVAC and D‐GC yielded an OR of 1.38 (95% CI 0.91–2.07; *p* = 0.13), indicating no significant difference in achieving pCR between the interventions. Treatment ranking based on *P*‐scores for pCR showed the highest probability for D‐GC (0.968), followed by ddMVAC (0.426) and GC (0.106). Heterogeneity was low (*τ* ^2^ = 0, *I* ^2^ = 0%) and no inconsistency between designs was observed (*Q* = 0.82, *p* = 0.36).

### 3.4 Treatment‐Related Adverse Events

Regarding TRAEs of at least grade 3, neither D‐GC (OR 0.99, 95% CI 0.77–1.26; *p* = 0.92) nor ddMVAC (OR 0.86, 95% CI 0.64–1.15; *p* = 0.31) differed significantly from GC (Figure 2). Indirect comparison of D‐GC and ddMVAC yielded an OR of 1.15 (95% CI 0.78–1.69; *p* = 0.47), indicating no significant difference between the two regimens. Treatment ranking based on *P*‐scores for TRAE ≥ grade 3 suggested the lowest probability for ddMVAC (0.805), followed by D‐GC (0.388) and GC (0.307). No heterogeneity (*I* ^2^ = 0%) or between‐design inconsistency (*Q* = 0.21, *p* = 0.64) was observed.

### 3.5 Subgroup Analyses

Exploratory subgroup analyses within the NIAGARA trial were performed to explore the potential impact of baseline tumour burden and nodal status and are summarised in Figure S2. In patients with < pT3 disease, D‐GC versus GC yielded an OS HR of 0.89 (95% CI 0.43–1.85; *p* = 0.75) and a PFS HR of 0.81 (95% CI 0.52–1.26; *p* = 0.35), while the OR for pCR was 1.68 (95% CI 1.05–2.70; *p* = 0.031). In the N0 subgroup, D‐GC versus GC was associated with an OS HR of 0.75 (95% CI 0.65–0.86; *p* < 0.001), a PFS HR of 0.68 (95% CI 0.56–0.82; *p* < 0.001) and a pCR OR of 1.55 (95% CI 1.08–2.23; *p* = 0.019). Indirect comparisons of D‐GC versus ddMVAC in patients with < pT3 disease showed similar outcomes for OS (HR 1.18, 95% CI 0.54–2.56; *p* = 0.68), PFS (HR 1.12, 95% CI 0.68–1.85; *p* = 0.66) and pCR (OR 1.49, 95% CI 0.81–2.73; *p* = 0.20). In the N0 subgroup, indirect comparisons between D‐GC and ddMVAC likewise suggested comparable outcomes for OS (HR 0.99, 95% CI 0.74–1.34; *p* = 0.97), PFS (HR 0.94, 95% CI 0.69–1.28; *p* = 0.69) and pCR (OR 1.37, 95% CI 0.81–2.32; *p* = 0.24).

### 3.6 Simulated Benchmarking of EV‐P Against Current Neoadjuvant Standards

**FIGURE 3:**
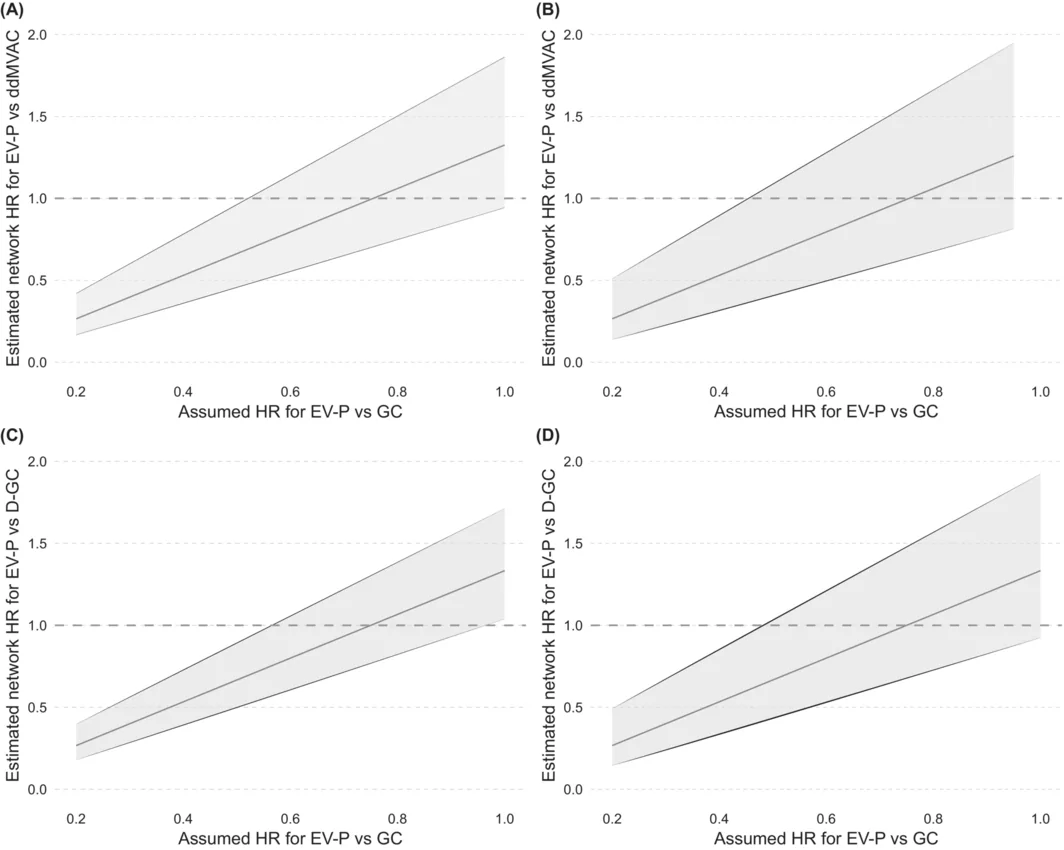
Simulation‐based network hazard ratios (HRs) for EV‐P versus ddMVAC and D‐GC at 2‐ and 5‐year timepoints. Panels (A and B) show results for the 2‐year follow‐up (A: EV‐P vs. ddMVAC; B: EV‐P vs. D‐GC), while Panels (C and D) display results for the 5‐year follow‐up (C: EV‐P vs. ddMVAC; D: EV‐P vs. D‐GC). The *x*‐axis reflects the assumed direct HR of EV‐P versus GC (range: 0.20–1.00), and the *y*‐axis shows the resulting network HR from indirect comparisons via GC. Solid lines represent point estimates; shaded areas indicate 95% confidence intervals (CIs). The dashed line marks the null effect (HR = 1.0). At 2 years, statistical superiority of EV‐P was achieved at assumed HRs ≤ 0.45 for both comparators; at 5 years, the thresholds increased to ≤ 0.50 for ddMVAC and ≤ 0.55 for D‐GC.

As shown in Figure 3, network HRs for EV‐P versus ddMVAC (Panel A and C) and D‐GC (Panel B and D) increased with less favourable assumed effects of EV‐P versus GC. EV‐P would only demonstrate a significant improvement in OS compared to ddMVAC at the 2‐year follow‐up if the assumed HR versus GC is ≤ 0.45. The same HR threshold applied to D‐GC. At the 5‐year follow‐up, statistical significance would be maintained for HRs of ≤ 0.50 versus ddMVAC and ≤ 0.55 versus D‐GC. Across all scenarios, these thresholds were associated with statistically significant outcomes, as indicated by *p*‐values ≤ 0.05 and upper confidence limits remaining below 1.0.

### 3.7 Risk of Bias and Certainty of Evidence

**FIGURE 4:**
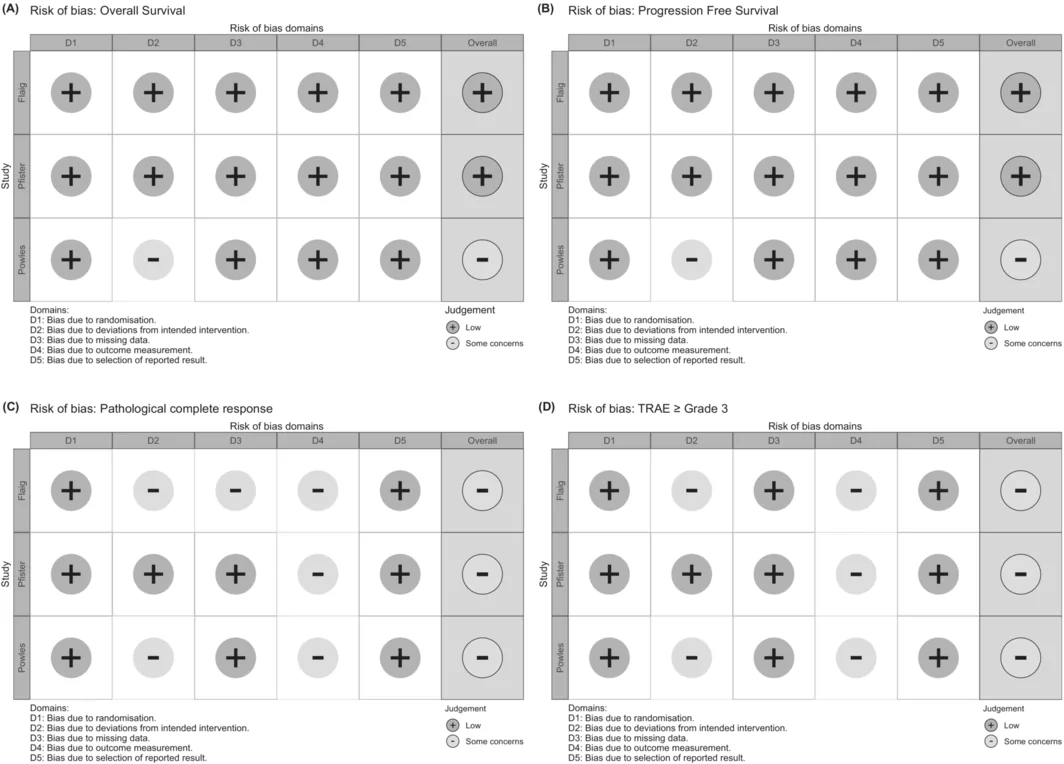
Risk of bias assessment using the RoB 2 tool for (A) overall survival, (B) progression‐free survival, (C) pathological complete response and (D) treatment‐related adverse events ≥ grade 3. Rows represent individual trials. Dark grey circles (+) indicate low risk of bias; light grey circles (−) indicate some concerns.

This section provides the GRADE assessment and Summary of Findings (SoF) table for the indirect comparison between D‐GC and ddMVAC (Table 2). GRADE assessments for the direct comparisons (ddMVAC vs. GC and D‐GC vs. GC) are provided in the Supporting Information (Tables S1 and S2). According to the GRADE assessment, the certainty of evidence for the indirect comparison was rated as moderate for the OS, PFS and pCR events (Table 2). This was primarily due to imprecision, reflected by wide confidence intervals of the indirect comparison from the NMA that crossed the line of no effect. Furthermore, the certainty of evidence regarding TRAE was rated as low due to severe imprecision. The Risk of Bias (RoB) assessment is illustrated in a traffic light plot (Figure 4).

**TABLE 2:** Summary of findings and GRADE assessment for the network meta‐analysis.

D‐GC compared to ddMVAC for neoadjuvant therapy prior to RC					
*Patient or population*: neoadjuvant therapy prior to RC					
*Intervention*: D‐GC					
*Comparison*: ddMVAC					
Outcomes	No. of participants (studies)	Certainty of the evidence (GRADE)	Relative effect (95% CI)	Anticipated absolute effects	
				Risk with ddMVAC	Risk difference with D‐GC*
Overall Survival (OS) assessed with: HR	913 (3 RCTs)	⨁⨁⨁◯ Moderate^a^	HR 0.99 (0.75–1.33) [Overall Survival]	330 per 1.000	2 fewer per 1.000 (from 71 fewer to 83 more)
Progression free survival (PFS) assessed with: HR	913 (3 RCTs)	⨁⨁⨁◯ Moderate^a^	HR 0.94 (0.69–1.28) [Progression free survival]	356 per 1.000	17 fewer per 1.000 (from 94 fewer to 75 more)
Pathological complete response (pCR) assessed with: OR	893 (3 RCTs)	⨁⨁⨁◯ Moderate^a^	OR 1.38 (0.91–2.07)	314 per 1.000	73 more per 1.000 (from 20 fewer to 173 more)
Treatment‐related adverse events ≥ 3 (TRAE) assessed with: OR	887 (3 RCTs)	⨁⨁◯◯ Low^a^	OR 1.15 (0.78–1.69)	476 per 1.000	35 more per 1.000 (from 61 fewer to 130 more)

*Note:* **GRADE Working Group grades of evidence**: *High certainty*: we are very confident that the true effect lies close to that of the estimate of the effect. *Moderate certainty*: we are moderately confident in the effect estimate: the true effect is likely to be close to the estimate of the effect, but there is a possibility that it is substantially different. *Low certainty*: our confidence in the effect estimate is limited: the true effect may be substantially different from the estimate of the effect. *Very low certainty*: we have very little confidence in the effect estimate: the true effect is likely to be substantially different from the estimate of effect.

Abbreviations: CI, confidence interval; HR, hazard ratio; OR, odds ratio; OS, overall survival; pCR, pathological complete response; PFS, progression‐free survival; TRAEs, treatment‐related adverse events.

^a^ Wide confidence intervals.

* The risk in the intervention group (and its 95% confidence interval) is based on the assumed risk in the comparison group and the relative effect of the intervention (and its 95% CI).

## 4 Discussion

This NMA aimed to address the lack of direct comparative evidence between neoadjuvant ddMVAC and perioperative D‐GC for the treatment of MIBC. Our findings demonstrate that both treatments significantly improve OS and PFS compared to standard GC, with no significant differences observed in the indirect NMA comparison between ddMVAC and D‐GC for either OS or PFS. D‐GC was associated with a higher likelihood of achieving pCR compared to GC, while ddMVAC showed no such advantage. The safety profiles of both intensified regimens were comparable to GC in terms of TRAEs ≥ grade 3. Overall, our findings confirm the oncological superiority of both intensified strategies over the previous standard of care, GC and support their clinical equivalence in terms of efficacy. Simulation‐based indirect NMA comparisons further indicated that EV‐P needs to demonstrate a substantial benefit over GC in order to show superiority over ddMVAC and D‐GC.

One important clinical question examined in this NMA is whether ddMVAC improves oncological outcomes compared to GC in the neoadjuvant setting. While the differences between conventional MVAC and GC have been extensively explored [20, 21], VESPER and SWOG 1314 have challenged the position of GC as the SOC. However, it remains unclear whether the observed benefit of ddMVAC relies on the higher cumulative cisplatin dose or on the combination of agents used in this regimen. This question is further highlighted by the differences in results in the included ddMVAC RCTs. In the VESPER trial, six cycles of ddMVAC led to a significant improvement in OS, whereas in the SWOG S1314 trial, which administered only four cycles of ddMVAC, no statistically significant OS benefit was detected. However, this lack of significance in SWOG S1314 may also be attributable to the lower statistical power of the trial. This, along with the historical concerns regarding the toxicity associated with MVAC‐based regimens, has likely contributed to the slow adoption of ddMVAC in clinical practice [22, 23]. Instead of intensifying the NAC regimen, an alternative strategy is to augment chemotherapy with immunotherapy. This approach was investigated in the NIAGARA trial, in which cisplatin‐eligible patients with operable MIBC were randomised to receive neoadjuvant GC alone or combined with durvalumab, followed by RC [8]. Patients in the intervention arm additionally received perioperative durvalumab for up to 1 year after RC. After a median follow‐up period of 3.8 years, the OS rate in the durvalumab group was significantly higher. It is noteworthy that patients receiving durvalumab had significantly higher rates of pCR. The rising rate of pCR patients has led to a debate of enabling a RC‐free treatment of patients with MIBC, which would indicate a paradigm shift. This is supported by the event‐free survival rates of 92% (95% CI, 87%–95%) and overall survival rates of 96% (95% CI, 92%–98%) in the pCR group in the D‐GC group of the NIAGARA trial [24]. However, while ctDNA has shown promise, the identification of these patients will pose a significant challenge in future trials and will likely involve some sort of combination of cystoscopy, ctDNA and possible urinary tumour DNA (utDNA) [25]. RC‐free strategies therefore remain strictly investigational outside of clinical trials.

From a clinical perspective, NAC in MIBC pursues three complementary goals: achieving pCR as the optimal oncological outcome, eliminating potential micrometastases and maintaining a perioperative safety profile that ensures patients remain fit for RC and, if residual disease is present, for prompt initiation of adjuvant chemo‐immunotherapy. This indirect comparison in this NMA informs the relative efficacy of ddMVAC and D‐GC as neoadjuvant strategies in the absence of head‐to‐head data. Our results demonstrated no statistically significant differences between ddMVAC and D‐GC regarding OS, PFS, or pCR. For OS, the NMA *P*‐scores of 0.758 for D‐GC and 0.733 for ddMVAC support this finding, indicating comparable treatment rankings. However, for pCR, the difference in *P*‐scores was more pronounced, with D‐GC ranking notably higher than ddMVAC (0.968 vs. 0.426). Although not statistically significant, the divergence in *P*‐scores suggests that D‐GC might be more effective in achieving pCR and, given its known survival benefit, may therefore be clinically favoured, irrespective of PD‐L1 expression status, as comparable effect sizes were observed across PD‐L1‐high and PD‐L1‐low/negative subgroups in the NIAGARA trial. These findings should be interpreted in light of relevant differences in baseline patient characteristics across trials. The NIAGARA trial, which evaluated D‐GC, enrolled a higher proportion of patients with > T2 disease and also included a small number of cN1 patients, which was unique among the included studies. To address the concern that this enrichment of locally advanced disease might bias our results in favour of D‐GC, we performed subgroup analyses within the NIAGARA trial, restricting the comparison of D‐GC versus GC to patients with < pT3 and to those with pN0 disease (Figure S2). Although point estimates in the lower‐risk subgroups were attenuated, the wide confidence intervals were compatible with the overall effect and the data did not support a statistically robust difference in treatment effect by T‐stage or nodal status. Thus, the available data do not support the hypothesis that the apparent benefit of D‐GC over GC is only confined to patients with locally advanced or node‐positive disease. Lastly, comparable stage‐ and node‐stratified estimates were not available for VESPER or SWOG S1314, so residual confounding by differences in tumour burden between trials cannot be excluded. From a patient selection perspective, these findings may support a risk‐adapted approach: D‐GC and ddMVAC may be preferable for patients with higher tumour burden or aggressive disease biology in whom maximising pCR probability is the priority, while GC may represent a more suitable option for patients in whom the specific toxicity profile of intensified regimens is a concern. The choice between the two intensified regimens may further be informed by regimen‐specific toxicity patterns, which are discussed in detail below.

When interpreting the results, it should also be noted that survival data in this analysis could not be analysed separately for purely neoadjuvant and perioperative cohorts. Moreover, the included trials differ substantially in design and patient populations. While SWOG was a phase II trial with the exploratory COXEN score as the primary endpoint, the other trials were phase III studies with survival‐related endpoints. In addition, treatment allocation also varied across studies: in the VESPER trial, chemotherapy could be administered in either the neoadjuvant or adjuvant setting, while in NIAGARA only neoadjuvant therapy was investigated. Differences in eligibility criteria, such as renal function (glomerular filtration rate ≥ 50 mL/min in VESPER vs. ≥ 40 mL/min in NIAGARA) and cardiac function (left ventricular ejection fraction ≥ 50% required in VESPER but not in NIAGARA), further highlight the heterogeneity between study populations. Furthermore, other adjuvant treatment options, such as nivolumab, have demonstrated survival benefits in high‐risk MIBC and may provide additional benefit following both GC and ddMVAC in the neoadjuvant setting [26]. However, due to the adjuvant‐only trial designs, these approaches could not be included in the present NMA.

The clinical applicability of these intensified regimens also depends on their safety and tolerability, an aspect that is crucial to patients and linked to adherence to therapy [27, 28]. This is underscored by the fact that, despite evidence indicating improved oncological outcomes with ddMVAC, GC continues to be widely used in clinical practice, largely due to its favourable toxicity profile and reduced treatment burden [20]. Accordingly, the comparative assessment of TRAEs represents another critical objective of this NMA. Our data show that there is no statistically significant difference in the overall rate of TRAEs ≥ grade 3 across the included treatments. However, data from the VESPER trial demonstrated that gastrointestinal toxicity and asthenia were significantly more common in the ddMVAC group [29]. In particular, asthenia is one of the most severe and disabling symptoms reported by patients undergoing chemotherapy despite often being considered mild by clinicians [30]. Moreover, only 58% of patients completed all six planned cycles of ddMVAC, compared to approximately 84% in the GC arm [17]. Although the patient‐specific reasons for treatment discontinuation were not documented, this may serve as an additional indirect indicator of reduced tolerability. Furthermore, the VESPER trial exclusively enrolled patients deemed eligible for ddMVAC, which may itself constitute a potential source of confounding.

The indirect comparison showed no significant differences in grade ≥ 3 TRAEs between D‐GC and ddMVAC. Nevertheless, clinically relevant differences in specific toxicity patterns may still inform individual regimen choice—for example, despite comparable overall toxicity rates in VESPER, ddMVAC was associated with markedly higher rates of severe asthenia, which may preclude its use in patients with limited physiological reserve.

It is important to note that toxicity assessments remain challenging in this NMA due to the considerable variation in the reporting of adverse events across trials. In the VESPER trial, toxicity was assessed and graded before each chemotherapy cycle according to the NCI Common Terminology Criteria for Adverse Events (CTCAE). In the NIAGARA trial, adverse events were systematically collected from the time of informed consent until 90 days after the last dose. All serious adverse events had to be reported within 24 h of the investigator becoming aware of them. Differences in treatment protocols, including the number of cycles (e.g., six vs. four neoadjuvant cycles of ddMVAC in VESPER vs. SWOG S1314), and the use of adjuvant therapy, also complicate direct comparisons, as it is not possible to derive a dose‐dependent toxicity profile. Unfortunately, this NMA lacks patient‐reported outcomes such as quality of life (QoL) during chemotherapy, yet these aspects would be crucial in this debate and could substantially inform therapeutic decision‐making.

EV‐P has demonstrated remarkable efficacy in metastatic urothelial carcinoma and is currently being explored as a potentially transformative antibody‐drug conjugate (ADC) option in the neoadjuvant/perioperative setting in the EV‐304 trial. While initially evaluated in cisplatin‐ineligible patients with advanced disease, its role in earlier‐stage treatment has gained increasing attention, particularly following promising early data from the EV‐103 trial. In this study, pCR rates of approximately 35% were achieved in cisplatin‐ineligible patients undergoing neoadjuvant therapy, comparable to conventional cisplatin‐based regimens [31, 32]. Our analysis showed that EV‐P needs to reach a HR ≤ 0.45 to be statistically superior to both ddMVAC and D‐GC at 2 years, with slightly more lenient thresholds at 5 years (HR ≤ 0.50 vs. ddMVAC and ≤ 0.55 vs. D‐GC). These results provide a clinically meaningful benchmark against which future trial outcomes can be interpreted, as it seems unlikely that EV‐P will be compared to ddMVAC or D‐GC in a direct comparison.

While EV‐P represents a highly attractive ADC strategy, toxicity remains a relevant concern. In early‐phase trials, TRAE ≥ grade 3 were reported in up to 55% of patients, including neuropathy (13%), rash (12%) and fatigue (7%) [33]. Additionally, rare, severe immune‐related toxicities have also been described, including one fatal case of Stevens‐Johnson syndrome [34]. Beyond clinical toxicity, EV‐P is also associated with a significant economic burden [35]. Assuming an average of nine treatment cycles, the total cost per patient could exceed $400,000 [36]. This raises the critical question of what magnitude of survival benefit would justify widespread use of EV‐P in an unselected patient population, especially when cost‐effectiveness and toxicity must be weighed against established, more affordable standard regimens. In summary, the role of EV‐P in the perioperative setting remains investigational, and our use of simulated HRs is intended to be hypothesis‐generating. An integration of EV‐P into neoadjuvant concepts for cisplatin‐eligible MIBC patients should therefore be restricted to carefully selected, well‐defined high‐risk populations within the context of larger clinical trials and dedicated subgroup analyses.

This study is not free of limitations. Although statistical heterogeneity was low, differences in trial design and treatment protocols and baseline patient characteristics may additionally challenge the assumption of transitivity. In addition, the lack of QoL data make it difficult to derive clear recommendations for action when oncological treatment options are statistically equivalent. The simulation of EV‐P efficacy is inherently hypothetical and based on assumed HRs and does not account for patient‐specific factors such as comorbidities, treatment adherence or toxicity profiles, which could substantially influence real‐world outcomes. Finally, the limited number of included RCTs restricted the robustness and increased the susceptibility to bias.

## 5 Conclusion

This NMA synthesises current evidence from randomised trials comparing the use of ddMVAC, D‐GC and GC in the neoadjuvant treatment of MIBC. Both ddMVAC and D‐GC demonstrated superior survival outcomes compared to standard GC, with no significant difference between ddMVAC and D‐GC in the indirect comparison. Simulated HR data suggest that EV‐P must achieve substantial survival benefit over GC in the neoadjuvant setting to outperform existing regimens. Together, these findings support evidence‐based treatment selection in MIBC and offer a reference framework for interpreting future trial results.

## Author Contributions

**Maurin Helen Mangold:** methodology, validation, writing – original draft, formal analysis. **Gloria Baumann:** methodology, writing – review and editing. **Victoria Luise Simone Wieland:** methodology, writing – review and editing. **Nicolas Carl:** writing – review and editing, formal analysis. **Luisa Vivienne Renner:** writing – review and editing. **Alexander Studier‐Fischer:** writing – review and editing. **Hanna Menold:** writing – review and editing. **Stefanie Zschäbitz:** supervision, writing – review and editing, conceptualization. **Frederik Wessels:** writing – review and editing. **Niklas Westhoff:** writing – review and editing, supervision. **Maurice Stephan Michel:** writing – review and editing, resources. **Karl‐Friedrich Kowalewski:** writing – review and editing, formal analysis, supervision, methodology. **Caelán Max Haney‐Aubert:** writing – review and editing, supervision, writing – original draft, methodology.

## Conflicts of Interest

The authors declare no conflicts of interest.

## Supporting information


**Figure S1:** Network structure of network meta‐analysis.
**Figure S2:** Forest plots for overall survival (OS, panel A), progression‐free survival (PFS, panel B) and pathological complete response (pCR, panel C).
**Table S1:** Summary of findings and GRADE assessment for the network meta‐analysis.
**Table S2:** Summary of findings and GRADE assessment for the network meta‐analysis.

## Data Availability

The data that support the findings of this study are available from the corresponding author upon reasonable request.
