# Supplementary material for: Indirect Comparison of Neoadjuvant Treatment Strategies for Muscle‐Invasive Bladder Cancer: ddMVAC and Perioperative Durvalumab‐Gemcitabine‐Cisplatin Versus Gemcitabine‐Cisplatin: A Systematic Review and Network Meta‐Analysis of Randomised Controlled Trials
Source: Int J Cancer. 2026 Apr 28;159(8):1962–73. doi: 10.1002/ijc.70525 (PMC13495798; doi:10.1002/ijc.70525)
Supplement: Supplementary file 1 [file IJC-159-1962-s001.pdf]

Supplementary Material for

Indirect Comparison of Neoadjuvant Treatment Strategies for Muscle-Invasive Bladder Cancer: ddMVAC and Perioperative Durvalumab-Gemcitabine-Cisplatin versus Gemcitabine-Cisplatin: A Systematic Review and Network Meta-Analysis of Randomized Controlled Trials

Maurin Helen Mangold, Gloria Baumann, Victoria Luise Simone Wieland, Nicolas Carl, Luisa Vivienne Renner, Alexander Studier-Fischer, Hanna Menold, Stefanie Zschäbitz, Frederik Wessels, Niklas Westhoff, Maurice Stephan Michel, Karl-Friedrich Kowalewski, Caelán Max Haney-Aubert

Table of contents:

Supplementary Figures S1 – S2

Supplementary Tables S1 – S2

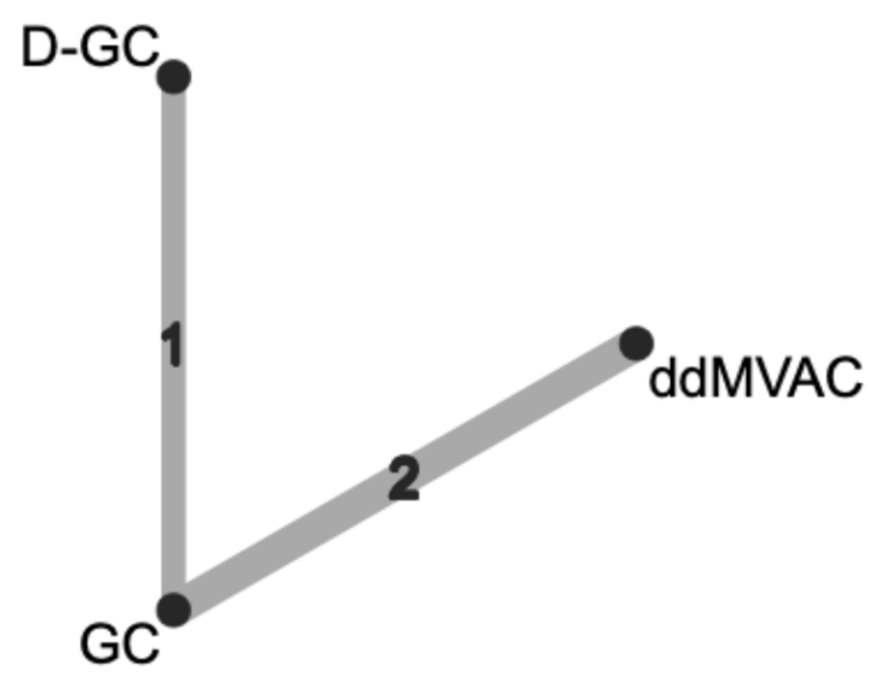

Supplementary Figure S1: Network structure of network meta-analysis. The diagram displays the evidence structure of the network meta-analysis (NMA). The three interventions are shown: dose-dense methotrexate, vinblastine, doxorubicin, and cisplatin (ddMVAC), Durvalumab plus gemcitabine cisplatin (D-GC), and gemcitabine cisplatin (GC). Lines in between interventions indicate direct pairwise comparisons; line thickness reflects the number of contributing studies. Two designs and three pairwise comparisons were included in the network. This network structure applies to all outcomes evaluated.

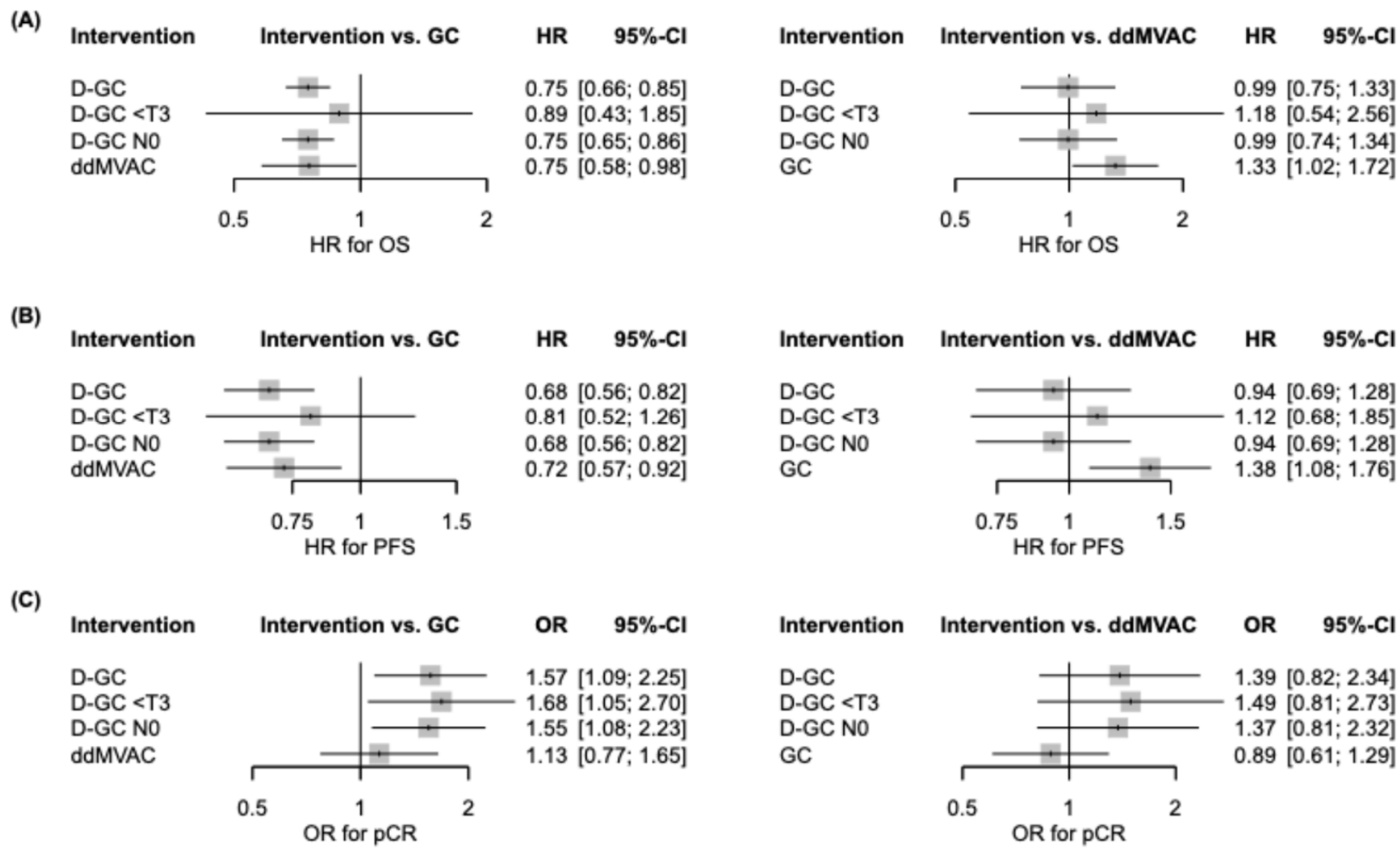

Supplementary Figure S2: Forest plots for overall survival (OS, panel A), progression-free survival (PFS, panel B), and pathological complete response (pCR, panel C). Each panel displays two network meta-analysis forest plots: interventions versus GC (left) and versus ddMVAC (right). Treatments shown include D-GC restricted to the overall population, to patients with clinical stage ≤T2 disease (<T3), and to clinically lymph node-negative patients (N0). Squares represent point estimates; horizontal lines represent 95% confidence intervals. The vertical reference line indicates no treatment effect (HR or OR = 1.0). ddMVAC, dose-dense methotrexate, vinblastine, doxorubicin, and cisplatin; D-GC, durvalumab plus gemcitabine-cisplatin; GC, gemcitabine-cisplatin; HR, hazard ratio; OR, odds ratio; CI, confidence interval.

Supplementary Table S1: Summary of Findings and GRADE Assessment for the network meta-analysis. TRAEs, treatment-related adverse events; OS, overall survival; PFS, progression-free survival; pCR, pathological complete response; HR, hazard ratio; OR, odds ratio; CI, confidence interval.

DdMVAC compared to GC for neoadjuvant therapy prior to RC

Patient or population: neoadjuvant therapy prior to RC
Intervention: ddMVAC
Comparison: GC

Outcomes	№ of participants (studies)	Certainty of the evidence (GRADE)	Relative effect (95% CI)	Anticipated absolute effects	
				Risk with GC	Risk difference with ddMVAC
Overall survival (OS) assessed with: HR	1249 (3 RCTs)	⨁⨁⨁⨁ High	**HR 0.75** (0.58 to 0.98) [Overall survival]	348 per 1.000	**74 fewer per 1.000** (from 128 fewer to 6 fewer)
Progression Free Survival (PFS) assessed with: HR	1249 (3 RCTs)	⨁⨁⨁⨁ High	**HR 0.72** (0.57 to 0.92) [Progression Free Survival]	460 per 1.000	**102 fewer per 1.000** (from 164 fewer to 27 fewer)
Pathological complete response (pCR) assessed with: OR	1250 (3 RCTs)	⨁⨁⨁◯ Moderate	**OR 1.14** (0.83 to 1.56)	282 per 1.000	**27 more per 1.000** (from 36 fewer to 98 more)
Treatment-related adverse events ≥ 3 (TRAE) assessed with: OR	1242 (3 RCTs)	⨁⨁⨁◯ Moderate[a]	**OR 0.86** (0.64 to 1.15)	451 per 1.000	**37 fewer per 1.000** (from 106 fewer to 35 more)

***The risk in the intervention group** (and its 95% confidence interval) is based on the assumed risk in the comparison group and the **relative effect** of the intervention (and its 95% CI).

CI: confidence interval; **HR:** hazard ratio; **OR:** odds ratio

GRADE Working Group grades of evidence
High certainty: we are very confident that the true effect lies close to that of the estimate of the effect.
Moderate certainty: we are moderately confident in the effect estimate: the true effect is likely to be close to the estimate of the effect, but there is a possibility that it is substantially different.
Low certainty: our confidence in the effect estimate is limited: the true effect may be substantially different from the estimate of the effect.
Very low certainty: we have very little confidence in the effect estimate: the true effect is likely to be substantially different from the estimate of effect.

Explanations
a. wide confidence intervals

Supplementary Table S2: Summary of Findings and GRADE Assessment for the network meta-analysis. TRAEs, treatment-related adverse events; OS, overall survival; PFS, progression-free survival; pCR, pathological complete response; HR, hazard ratio; OR, odds ratio; CI, confidence interval.

D-GC compared to GC for neoadjuvant therapy prior to RC

Patient or population: neoadjuvant therapy prior to RC
Intervention: D-GC
Comparison: GC

Outcomes	№ of participants (studies)	Certainty of the evidence (GRADE)	Relative effect (95% CI)	Anticipated absolute effects	
				Risk with GC	Risk difference with D-GC
Overall survival (OS) assessed with: HR	1422 (3 RCTs)	⊕⊕⊕⊕ High	**HR 0.75** (0.66 to 0.85) [Overall survival]	348 per 1.000	**74 fewer per 1.000** (from 102 fewer to 43 fewer)
Progression free survival (PFS) assessed with: HR	1422 (3 RCTs)	⊕⊕⊕⊕ High	**HR 0.68** (0.56 to 0.82) [Progression free survival]	460 per 1.000	**118 fewer per 1.000** (from 168 fewer to 63 fewer)
Pathological complete response (pCR) assessed with: OR	1423 (3 RCTs)	⊕⊕◯◯ Low[a]	**OR 1.57** (1.21 to 2.03)	282 per 1.000	**99 more per 1.000** (from 40 more to 162 more)
Treatment-related adverse events ≥ 3 (TRAE) assessed with: OR	1415 (3 RCTs)	⊕⊕⊕◯ Moderate[a]	**OR 0.99** (0.77 to 1.26)	451 per 1.000	**2 fewer per 1.000** (from 64 fewer to 58 more)

***The risk in the intervention group** (and its 95% confidence interval) is based on the assumed risk in the comparison group and the **relative effect** of the intervention (and its 95% CI).

CI: confidence interval; **HR:** hazard ratio; **OR:** odds ratio

GRADE Working Group grades of evidence
High certainty: we are very confident that the true effect lies close to that of the estimate of the effect.
Moderate certainty: we are moderately confident in the effect estimate: the true effect is likely to be close to the estimate of the effect, but there is a possibility that it is substantially different.
Low certainty: our confidence in the effect estimate is limited: the true effect may be substantially different from the estimate of the effect.
Very low certainty: we have very little confidence in the effect estimate: the true effect is likely to be substantially different from the estimate of effect.

Explanations

a. wide confidence intervals
